# Predictive diagnosis of the risk of breast cancer recurrence after surgery by single-particle quantum dot imaging

**DOI:** 10.1038/srep14322

**Published:** 2015-09-22

**Authors:** Kohsuke Gonda, Minoru Miyashita, Hideo Higuchi, Hiroshi Tada, Tomonobu M. Watanabe, Mika Watanabe, Takanori Ishida, Noriaki Ohuchi

**Affiliations:** 1 Department of Medical Physics, Graduate School of Medicine, Tohoku University, Seiryo-machi, Aoba-ku, Sendai 980-8575, Japan; 2 Department of Nano-Medical Science, Graduate School of Medicine, Tohoku University, Seiryo-machi, Aoba-ku, Sendai 980-8575, Japan; 3 Department of Surgical Oncology, Graduate School of Medicine, Tohoku University, Seiryo-machi, Aoba-ku, Sendai 980-8574, Japan; 4 Department of Physics, Graduate School of Science, University of Tokyo, Hongo Bunkyou-ku Tokyo 113-0033, Japan; 5 Laboratory for Comprehensive Bioimaging, RIKEN Quantitative Biology Center, 6-2-3, Furuedai, Suita, Osaka 565-0874, Japan; 6 Department of Pathology, Tohoku University Hospital, Seiryo-machi, Aoba-ku, Sendai 980-8574, Japan

## Abstract

In breast cancer, the prognosis of human epidermal growth factor receptor 2 (HER2)-positive patients (20–25%) has been dramatically improved by the clinical application of the anti-HER2 antibody drugs trastuzumab and pertuzumab. However, the clinical outcomes of HER2-negative cases with a poor prognosis have not improved, and novel therapeutic antibody drugs or diagnostic molecular markers of prognosis are urgently needed. Here, we targeted protease-activated receptor 1 (PAR1) as a new biomarker for HER2-negative patients. The developed anti-PAR1 antibody inhibited PAR1 activation by matrix metalloprotease 1 and thereby prevented cancer-cell migration and invasion. To estimate PAR1 expression levels in HER2-negative patient tissues using the antibody, user-friendly immunohistochemistry with fluorescence nanoparticles or quantum dots (QDs) was developed. Previously, immunohistochemistry with QDs was affected by tissue autofluorescence, making quantitative measurement extremely difficult. We significantly improved the quantitative sensitivity of immunohistochemistry with QDs by using an autofluorescence-subtracted image and single-QD imaging. The immunohistochemistry showed that PAR1 expression was strongly correlated with relapse-free survival time in HER2-negative breast cancer patients. Therefore, the developed anti-PAR1 antibody is a strong candidate for use as an anticancer drug and a prognostic biomarker for HER2-negative patients.

Metastasis is the critical event in the prognosis of cancer patients and is a complex and interconnected multiple-step process^1^,^2^. Breast cancer is the major cause of cancer death among women worldwide, and approximately one-third of patients eventually develop metastasis^3^,^4^. Twenty to twenty-five percent of patients with breast cancer display an overexpression of human epidermal growth factor receptor 2 (HER2)/neu in their tumors. HER2-positive status is correlated with aggressive and poorly differentiated tumors and results in a worse prognosis^5^,^6^. Trastuzumab is a humanized monoclonal antibody against the HER2 protein and improves clinical outcomes for these patients^7^,^8^,^9^. Recently, in addition to trastuzumab, the new anticancer drugs pertuzumab and trastuzumab-emtansine were developed against HER2^10^,^11^. However, 75-80% of patients with breast cancer are negative for HER2. In addition to HER2, estrogen receptor (ER) and progesterone receptor (PgR) were among the first biomarkers recommended for routine clinical use^12^. Patients who are negative for HER2, ER, and PgR are categorized as triple-negative cases, a status often associated with a poor prognosis resulting from other disease-causing factors and the ineffectiveness of therapy targeting HER2, ER, or PgR^13^,^14^. Such patients are therefore in desperate need of new drugs that target molecules other than HER2, ER, PgR, or their downstream proteins.

Antibody drugs work simultaneously as an inhibitor of the targeted protein’s function and a trigger of antibody-dependent cellular cytotoxicity. Drugs consisting of amino acids are thought to have lower toxicity compared with small-molecule chemical drugs. To discover new antibody drugs for HER2-negative breast cancer patients, it is important to determine which proteins are highly expressed in cancer tissues with a poor prognosis. In this study, we targeted protease-activated receptor 1 (PAR1) as a new biomarker in HER2-negative patients. PAR1 is a G protein-coupled receptor that plays an important role in metastatic processes in various cancers of the breast, colon, lung, pancreas, and prostate^15^,^16^,^17^. PAR1 expression is highly elevated in metastatic breast cancer cells compared with non-metastatic or normal breast epithelial cells^18^. Matrix metalloprotease 1 (MMP1) functions as a protease agonist of PAR1 and activates PAR1 by cleaving its exodomain at the Arg_41_–Ser_42_ peptide bond^19^. This activation of PAR1 promotes cell migration and invasion. These results suggest that therapeutic blockade of MMP1 would provide a clinical benefit in the treatment of invasive breast cancer. However, numerous clinical trials of MMP1 inhibitors have suffered from results demonstrating dose-limiting toxicity^20^,^21^. Therefore, inhibition of the activity of PAR1 by directly targeting PAR1 itself, rather than MMP1, may be crucial to safely suppressing cancer-cell migration and invasion.

To estimate the effectiveness of an antibody drug against cancer, it is crucial that the expression level of the antibody-targeted protein in tumor cells is measured by immunohistochemistry (IHC). However, in IHC with 3,3′-diaminobenzidine (DAB) (IHC-DAB), the intensity of DAB staining depends on the enzymatic activity of horseradish peroxidase (HRP). Therefore, the staining intensity of DAB is significantly influenced by the reaction time, temperature and HRP substrate concentrations. The fluorescent label increases the quantitative sensitivity of IHC because the intensity of the fluorescent materials is proportional to the intensity of the photon excitation energy in an irreversible chemical reaction. Additionally, the fluorescent label provides an image with a high signal-to-noise ratio through the use of dark background light and multistaining with various wavelengths. In previous studies, a fluorescence imaging system was developed with Cy-5 tyramide for compartmentalized, automated, quantitative analysis of histological sections (AQUA)^22^,^23^. This method improved the quantitative sensitivity compared with IHC-DAB. However, general organic fluorescent molecules such as FITC, Alexa Fluors, and Cy-5, have disadvantages arising from their poor photostability and autofluorescence interference. In addition, the AQUA method obtains signal amplification via enzymatic activity. Therefore, AQUA method is less practical for clinical IHC with high quantitative sensitivity. Recently, quantum dots (QDs) have been used in various bio-imaging techniques due to their greater photostability and brightness compared with general organic fluorescent molecules. However, the high intensity of tissue autofluorescence is comparable to that of QDs. This problem has impeded quantitative analysis using only the fluorescence intensity of QDs in the presence of autofluorescence, as demonstrated by AQUA.

Here, we used fluorescence imaging with anti-PAR1 antibody-conjugated QDs (anti-PAR1-QDs) to demonstrate that migration and invasion by PAR1-expressing cancer cells were suppressed by the administration of an anti-PAR1 antibody. Furthermore, we developed a new IHC technique with quantitative sensitivity using QDs and image processing. The immunostaining results for HER2-negative human breast cancer tissue samples with anti-PAR1-QDs showed that the PAR1 expression level in cancer cells with a poor prognosis was strongly correlated with the prognosis of HER2-negative breast cancer patients.

## Results

### Inhibitory effect of monoclonal anti-PAR1 antibody on migration and invasion by breast cancer cells

**Figure 1:**
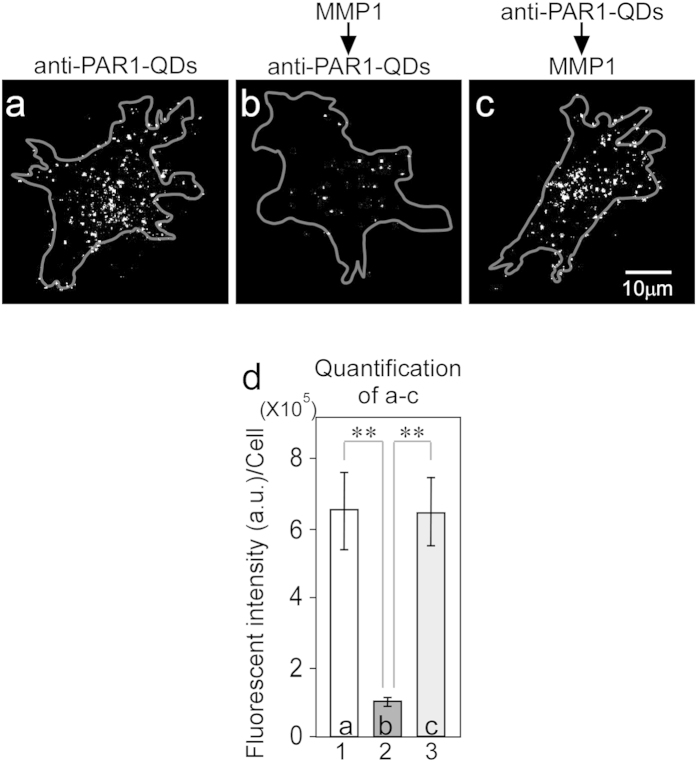
Monoclonal anti-PAR1 antibody abrogates the PAR1-cleaving activity of MMP1. (**a**–**c**) Fluorescence images showing the effect of anti-PAR1 antibody on the PAR1-cleaving activity of MMP1. In all of the cell images, anti-PAR1-QDs on the cell membrane located near the glass surface were visualized. The gray lines show the cellular outline delineated using bright-field images. The bright spots are the QD signals. Excitation wavelength (Ex), 488 nm; emission wavelength (Em), 695–740 nm. (**a**) PAR1-KPL cell treated with 40 nM anti-PAR1-QDs. (**b**) PAR1-KPL cell treated with 50 nM MMP1 and then with 40 nM anti-PAR1-QDs. (**c**) PAR1-KPL cell treated with 40 nM anti-PAR1-QDs and then with 50 nM MMP1. (**d**) The fluorescence intensities of QDs in a cell were calculated in the above three experiments. In each condition, the QD-fluorescence intensity of 11 cells in a cell were measured and calculated as gray values. Error bars indicate s.e.m. **indicates *P* < 0.01.

We previously developed a monoclonal anti-PAR1 antibody and successfully visualized the movements of PAR1-expressing cancer cells during metastasis *in vivo* by tracking cancer cells labeled with anti-PAR1-QDs, which possess 705-nm peak emission wavelength^24^. Epitope mapping of the anti-PAR1 antibody showed that it specifically reacted with N_35_ATLDPRSFLL_45_, including the R_41_-S_42_ peptide bond, which is the domain of PAR1 that is cleaved by MMP1. Therefore, binding between the anti-PAR1 antibody and PAR1 was anticipated to prevent PAR1 cleavage by MMP1. We previously developed PAR1-expressing KPL-4 cells (PAR1-KPL cells) by transfecting the PAR1 gene into the KPL-4 human breast cancer cell line. Three different experiments were performed to test the effect of anti-PAR1 antibody on the PAR1-cleaving activity of MMP1. The anti-PAR1-QDs did not bind to KPL-4 cells (data not shown) but specifically bound to PAR1 on the membrane of PAR1-KPL cells without MMP1 treatment (Fig. 1a). In all of the cell images in Fig. 1, anti-PAR1-QDs on the cell membrane located near the glass surface were visualized. Next, PAR1-KPL cells were treated with 50 nM MMP1 and then with 40 nM anti-PAR1-QDs. The anti-PAR1-QDs could not bind to PAR1 on the cell membrane because MMP1 cleaved the R_41_-S_42_ peptide bond of the N_35_ATLDPRSFLL_45_ sequence, which is an epitope of the anti-PAR1 antibody (Fig. 1b). Conversely, when PAR1-KPL cells were treated with 40 nM anti-PAR1-QDs and then with 50 nM MMP1, the anti-PAR1-QD interaction with membrane-bound PAR1 was maintained (Fig. 1c), as in Fig. 1a. The binding of anti-PAR1-QDs and PAR1 on the cell membrane was calculated by the fluorescence intensity of the QDs (Fig. 1d). These results suggest that the monoclonal anti-PAR1 antibody specifically interacts with PAR1 on the cell membrane in PAR1-KPL cells and inhibits PAR1-cleaving activity by MMP1.

**Figure 2:**
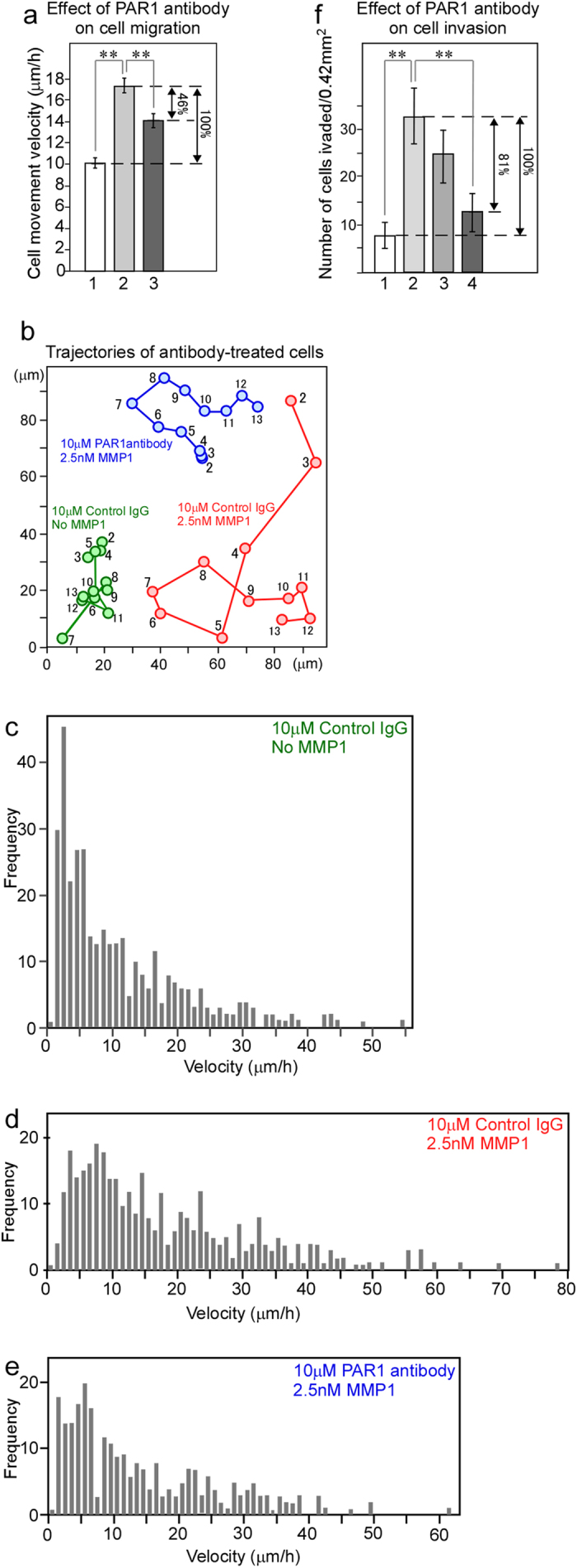
Monoclonal anti-PAR1 antibody inhibits PAR1-KPL cell migration and invasion activated by MMP1. (**a**) The effect of anti-PAR1 antibody/inhibitory activity on cell migration. Bar 1, migration velocity of cells treated with 10 μM control mouse IgG. Bar 2, velocity of cells treated with 10 μM control mouse IgG and then 2.5 nM MMP1. Bar 3, velocity of cells treated with 10 μM anti-PAR1 antibody and then 2.5 nM MMP1. In each condition, the nuclear barycentric positions of approximately 30 cells from 2–13-h video data were tracked, and the position of each cell every hour was calculated as the migration velocity (μm/h). (**b**) Typical patterns of the trajectories of antibody-treated PAR1-KPL cells. The numbers within the trajectories indicate the time (hours) after the start of the time-lapse imaging. (**c**) Histogram showing the velocity of PAR1-KPL cells treated with 10 μM control mouse IgG. (**d**) Histogram showing the velocity of PAR1-KPL cells treated with 10 μM control mouse IgG and then 2.5 nM MMP1. (**e**) Histogram showing the velocity of PAR1-KPL cells treated with 10 μM anti-PAR1 antibody and then 2.5 nM MMP1. The mean of all of the values in (**c**–**e**) are shown in bars 1, 2, and 3 in (**a**), respectively. (**f**) The effect of anti-PAR1 antibody-inhibitory activity on PAR1-KPL-cell invasion. Bar 1, the number of invading cells in a 0.42-mm^2^ area treated with 10 μM control mouse IgG. Bar 2, the number of invading cells in a 0.42-mm^2^ area treated with 10 μM control mouse IgG and then 2.5 nM MMP1. Bar 3, the number of invading cells in a 0.42-mm^2^ area treated with 3 μM anti-PAR1 antibody and then 2.5 nM MMP1. Bar 4, the number of invading PAR1-KPL cells in a 0.42-mm^2^ area treated with 10 μM anti-PAR1 antibody and then 2.5 nM MMP1. Error bars indicate s.e.m. **indicates *P* < 0.01.

Next, we investigated the effect of monoclonal anti-PAR1 antibody on the migration of PAR1-KPL cells. The PAR1-KPL cells display a cell-movement velocity of 10.2 μm/h on culture dishes without MMP1 treatment after incubation with 10 μM control mouse IgG (Fig. 2a, bar 1). The typical trajectory and a histogram of velocity are shown in Fig. 2b (green line) and 2c, respectively. When the PAR1-KPL cells were treated with 10 μM control mouse IgG and then with 2.5 nM MMP1, the movement velocity of the cells increased to 17.2 μm/h on culture dishes (Fig. 2a, bar 2). The typical trajectory and a histogram of velocity also revealed an increase in migration activity with MMP1 (Fig. 2b, red line and Fig. 2d). However, when the PAR1-KPL cells were treated with 10 μM anti-PAR1 antibody and then with 2.5 nM MMP1, the velocity decreased to 14.0 μm/h (Fig. 2a, bar 3). The typical trajectory and a histogram of velocity indicate a decrease in migration activity (Fig. 2b, blue line and Fig. 2e). Calculations were performed to estimate the inhibition of cell migration following treatment with 10 μM anti-PAR1 antibody. Fig. 2a shows that PAR1 antibody in the presence of MMP1 decreased velocity (17.2 [bar 2] – 14.0 [bar 3] = 3.2 μm/h) compared with MMP1 alone, which increases the number of cells (17.2 [bar 2]–10.2 [bar 1] = 7.0 μm/h) by 46% (3.2/7.0 × 100%), illustrating that PAR1 antibody inhibited cell migration activated by MMP1.

Next, the effect of monoclonal anti-PAR1 antibody on cell invasion was evaluated. In the cell-invasion assay on a matrigel-coated membrane, when the PAR1-KPL cells were treated with 10 μM control mouse IgG and not with MMP1, there were 8.0 invading cells per tested area (0.42 mm^2^) (Fig. 2f, bar 1). When the PAR1-KPL cells were treated with 10 μM control mouse IgG and then with 2.5 nM MMP1, the number of invading cells increased to 32.9 cells/0.42 mm^2^ (Fig. 2f, bar 2). However, when the PAR1-KPL cells were treated with 3 or 10 μM anti-PAR1 antibody and then with 2.5 nM MMP1, the number of invading cells decreased to 24.7 cells/0.42 mm^2^ (Fig. 2f, bar 3) or 12.8 cells/0.42 mm^2^ (Fig. 2f, bar 4), respectively. The following calculation was performed to estimate the inhibitory effect on cell invasion of treatment with 10 μM anti-PAR1 antibody. Figure 2f illustrates that PAR1 antibody in the presence of MMP1 resulted in a decreased number of cells (32.9 cells [bar 2]–12.8 cells [bar 4] = 20.1 cells/0.42 mm^2^) compared with the MMP1 alone, which increases the number of cells (32.9 cells [bar 2]–8.0 cells [bar 1] = 24.9 cells/0.42 mm^2^) was 81% (20.1/24.9 × 100%), indicating that PAR1 antibody inhibited cell invasion activated by MMP1. These results suggest that humanization of this anti-PAR1 antibody would provide an effective molecular target for an anticancer drug against PAR1.

### Imaging of single anti-PAR1-QD particles in human breast cancer tissues

A method to measure the expression level of the antibody-targeted protein in tumor cells is needed to estimate the response to the antibody drug in cancer patients with high accuracy. Although the application of QDs to IHC is highly anticipated, tissues possess a high intensity of autofluorescence that is comparable to that of QDs, which impedes quantitative analysis using only the fluorescence intensity of QDs in the presence of tissue autofluorescence. To address this problem, we developed new imaging techniques to evaluate differences between QD fluorescence and autofluorescence with high quantitative sensitivity by single-particle imaging.

**Figure 3:**
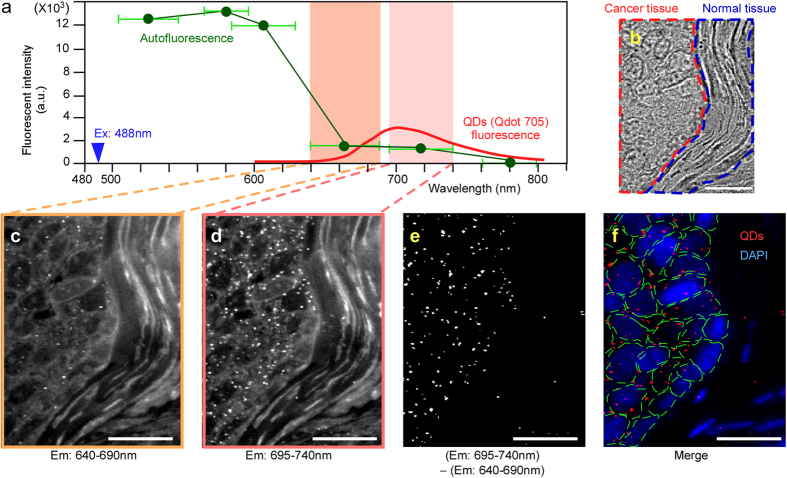
Development of single anti-PAR1-QD particle imaging of human breast cancer tissues. **(a**) The fluorescence-intensity curves for autofluorescence (green line) and QDs (red line) in 488-nm laser-excited tissues (blue arrowhead). To measure the autofluorescence pattern, the autofluorescence intensity was examined at various wavelengths in human breast cancer tissues using various bandpass filters (505–545 nm, 565–595 nm, 585–630 nm, 640–690 nm, 695–740 nm, and 760–800 nm). The fluorescence intensity at various wavelengths was divided by the wavelength interval. The averaged fluorescence intensity was plotted as the center value of the individual wavelengths, i.e., the calculated fluorescence intensity filtered with bandpass filters of 505–545 nm, 565–595 nm, 585–630 nm, 640–690 nm, 695–740 nm, and 760–800 nm were plotted at 525, 580, 607.5, 665, 717.5, and 780 nm, respectively. The horizontal green lines show the wavelength interval of each bandpass filter. The change in QD fluorescence was obtained from the Life Technologies website. The value of the QD-fluorescence interval ranging from 695–740 nm became 1.9-fold greater than that of the autofluorescence ranging from 695–740 nm. (**b**) Bright-field image of a human breast cancer tissue section including cancer (red area) and normal tissues (blue area). (**c**) The image filtered with the 640–690-nm bandpass filter. This image contains mainly the autofluorescence signal. (**d**) The image filtered with the 695–740-nm bandpass filter. This image contains the QD and autofluorescence signals. (**e**) The image after subtracting the 640–690-nm image (**c**) from the 695–740-nm image (**d**). Only the QD-fluorescence signals are visualized. **(f**) The merged image of QD signals (red spots) and nuclei of cancer cells (blue) stained with DAPI. The green lines indicate the cellular outline delineated using a bright-field image. Bar, 20 μm.

First, we investigated the change in the autofluorescence intensity of various wavelengths in human breast cancer tissues with various bandpass filters (505–545 nm, 565–595 nm, 585–630 nm, 640–690 nm, 695–740 nm, and 760–800 nm). The 488-nm laser-excited fluorescence intensity was measured in identical areas of the tissues. The total fluorescence intensity that passed through the filter was divided by the wavelength bandwidth of each filter. Comparison of the fluorescence intensity per unit wavelength interval showed that the autofluorescence intensity was high at less than 600 nm and decreased rapidly at wavelengths longer than 600 nm (Fig. 3a, green line). The change in QD fluorescence at the 705-nm peak emission wavelength is available from the Life Technologies website (Fig. 3a, red line). The fluorescence intensity of single-particle QD and autofluorescence in an arbitrary area (n = 15) of the same-sized region of interest (ROI) was compared using a 488-nm laser and 695–740-nm bandpass filters, and the intensity of single-particle QD fluorescence was 1.9-fold greater than that of the autofluorescence. The three datasets for the change in autofluorescence intensity, the change in single-QD-fluorescence intensity, and the comparison of autofluorescence and QD-fluorescence intensity are combined in Fig. 3a. These fluorescence patterns were used to develop new imaging techniques for IHC using an autofluorescence-subtracted image and single-QD imaging.

To estimate the fluorescence of QDs with high accuracy, the signal should be measured not as the fluorescence intensity, but as the QD particle number in a cell or a defined area in tissues. The fluorescence intensity of the QDs changes depending on the individual optical system and is affected by autofluorescence. Previous IHC techniques with QDs have used the fluorescence intensity of QDs, and it has thus been difficult to obtain a quantitative measurement with high accuracy^25^,^26^,^27^; however, the particle number of QDs is an absolute value and does not vary across systems or in the presence of autofluorescence. We developed the following new imaging techniques for IHC using an autofluorescence-subtracted image and single-particle QD imaging to accurately measure the particle number of QDs, excluding the effect of autofluorescence. First, deparaffinized human breast cancer tissue sections, including cancer and normal tissues (Fig. 3b), were immunostained with anti-PAR1-QDs using the IHC procedure. In the tissues excited with the 488-nm laser and filtered with a 695–740-nm bandpass filter (695–740-nm image), autofluorescence derived from the tissues and QD fluorescence were observed, and the anti-PAR1-QDs specifically bound to cancer tissues and not to normal tissues (Fig. 3d). By contrast, in the tissues excited with the 488 nm laser and filtered with a 640–690-nm bandpass filter (640-690-nm image), autofluorescence was clearly observed, but the intensity of the QD fluorescence was very low (Fig. 3c) because they have a 705-nm peak fluorescence intensity at a wavelength interval of 695–740 nm (Fig. 3a). Additionally, the pattern of autofluorescence in the 695–740-nm image was highly similar to that in the 640–690-nm image (Fig. 3a–d). Based on these results, to detect only QD fluorescence in 488-nm laser-excited tissues, we developed a method using an autofluorescence-subtracted image. We subtracted the 640–690-nm image mainly containing the autofluorescence signal from the 695–740-nm image containing both QD and autofluorescence signals, allowing us to visualize only the signal from the QDs (Fig. 3e). After we had created the subtracted image, we confirmed that the signal intensity of the autofluorescence in the 695–740-nm image was zero (Fig. 3e). Additionally, there was nearly no chromatic aberration in the subtracted image because the wavelengths of the 695–740-nm and 640–690-nm images are very close to one another.

**Figure 4:**
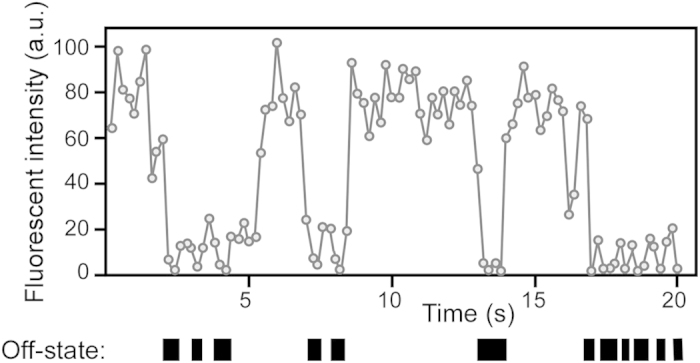
Typical fluorescence-intensity time traces of a single QD particle in 488 nm laser-excited breast cancer tissues. QD fluorescence consists of fluorescent and non-fluorescent states, called on and off states, respectively. We defined the fluorescence intensity below 10% of the maximum fluorescence intensity for each QD particle as its off state. Black boxes indicate the off state. When we measured the fluorescence of fresh QD particles and analyzed their properties, the results indicated that the mean time in the off state during 20 s of observation was approximately 4 s, with a low s.e.m. Therefore, based on an off-state time of 4 s, we selected a single-particle QD using each subtracted image and video image and then measured the fluorescence intensity of the single QD particle.

To calculate the number of QDs in the 695–740-nm image, we defined the fluorescence intensity of a single QD. Because QDs possessing the same fluorescent wavelength are uniform in size, the fluorescence intensity of a QD is proportional to the number of particles. QD fluorescence consists of fluorescent and non-fluorescent states called on and off states. This property results in blinking. When we measured the fluorescence of fresh QD particles and analyzed their properties, the results indicated that the mean time in the off state during a 20-s observation was approximately 4 s (Fig. 4), with an extremely low s.e.m. If several QDs are aggregated, the mean time in the off state per unit time is shortened because the on and off states of each particle in the aggregate occur randomly. Therefore, based on an off-state time of 4 s, we selected a single-particle QD using each autofluorescence-subtracted image and video image and then measured the fluorescence intensity of the particle (single-QD value). The cell number in each image was measured using the DAPI image (Fig. 3f). The total QD-fluorescence intensity was divided by the single-QD value. Then, the number of QD particles was divided by the number of cells to calculate the number of QD particles in a cell.

To determine the binding ratio between anti-PAR1-QDs and PAR1 on the cell membrane, we prepared anti-PAR1-QDs by mixing the monomerized monoclonal anti-PAR1 antibody (IgG) and QDs in a molar ratio of approximately 3:1. Then, the anti-PAR1-QDs were prepared using a Qdot 705 Antibody Conjugation Kit (Life Technologies) according to the manufacturer’s instructions, as in a previous study^28^. The diameters of the QD and monomer antibody are approximately 20 nm and 7–8 nm^29^, respectively, yielding a volume ratio of approximately 20:1. We estimated the number of monomer anti-PAR1 antibodies bound to the surface of a single QD by 0.8% agarose-gel electrophoresis, and the sample of anti-PAR1-QDs was fractionated into two major bands. Approximately 52% of the anti-PAR1-QDs were conjugated with three monomer antibody fragments and 48% with two fragments (mean value, 2.5 of monomer antibody fragments per single QD) (Supplementary Fig. 1). This data was similar to the result in our previous study^28^. These results demonstrate the following three features of anti-PAR1-QDs: (1) the monomer monoclonal anti-PAR1 antibody on anti-PAR1-QDs interacts with PAR1 on the cell membrane in a one-to-one interaction; (2) the volume of monomer PAR1 antibody is considerably smaller than that of QD; and (3) the monomer PAR1 antibody binds to the QD surface at very low density. Therefore, these data strongly suggest that anti-PAR1-QDs interact with a single PAR1 on the cell membrane. Using this new method, we successfully estimated the expression level of PAR1 as the particle number of anti-PAR1-QDs, rather than the fluorescence intensity.

### Prognostic prediction by IHC with anti-PAR1-QDs in HER2-negative human breast cancer

**Figure 5:**
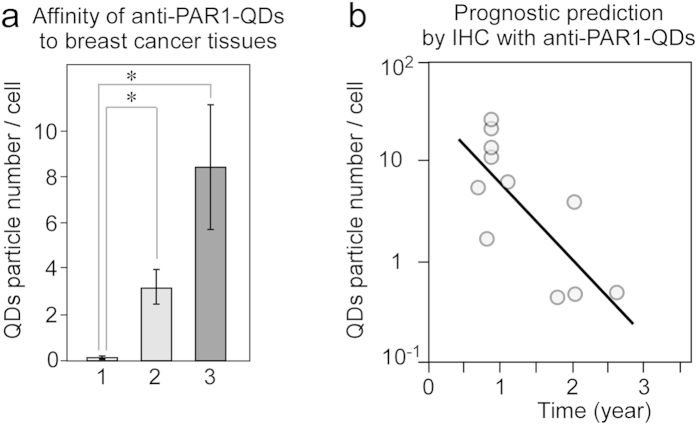
Development of prognostic prediction by IHC with anti-PAR1-QDs in human breast cancer. (**a**) A graph showing the affinity of anti-PAR1-QDs for human breast tissue. Bar 1, the number of anti-PAR1-QDs in a normal epithelial cell of the breast (n = 4 tissues). Bar 2, the number of anti-PAR1-QDs in a breast cancer cell representative of cases that remained non-metastatic for more than 5 years after surgery (n = 5 tissues). Bar 3, the number of anti-PAR1-QDs in a breast cancer cell representative of metastatic cases with recurrence within 3 years after surgery (n = 11 tissues). *indicates *P* < 0.05. The numbers of anti-PAR1-QDs specifically bound to the tissues from each patient are shown in Table 1. (b) A scatterplot diagram showing the number of anti-PAR1-QD particles in a metastatic cancer cell sample (**a**, bar 3) and relapse-free survival time (year) after surgery. The logarithmic y-axis shows the number of anti-PAR1-QD particles. The x-axis shows the relapse-free survival time (year) after surgery. The line graph shows the inverse correlation between these two factors. The correlation coefficient (R) is 0.75.

To apply IHC with anti-PAR1-QDs in a clinical setting, we selected four normal breast tissue samples from breast cancer patients, five HER2-negative breast cancer cases that remained non-metastatic for more than 5 years after surgery, and eleven HER2-negative cases with metastasis within 3 years after surgery, including seven triple-negative cases. Detailed clinical information is available for all of the cases. The patients with metastases were diagnosed as having a recurrence within 3 years after surgery at Tohoku University Hospital. After immunostaining with anti-PAR1-QDs, we calculated the number of QD particles in a cell by single-QD imaging. The clinical information and number of QD particles in each case are listed in Table 1. In normal epithelial cells of the breast, few QD particles were observed in the autofluorescence-subtracted images (Fig. 5a, bar 1). This result is supported by previous reports showing that normal breast epithelial cells express low levels of PAR1 protein^18^. The mean number of QD particles per cell in the breast cancer cases with metastasis was 8.4 (Fig. 5a, bar 3), versus 3.2 in non-metastatic cases (Fig. 5a, bar 2). Furthermore, we examined the relationship between the number of QD particles in a cell and relapse-free survival time (year) after surgery in eleven HER2-negative breast cancer cases with metastasis. The scatterplot diagram in Fig. 5b illustrates that these two factors were inversely correlated in metastatic cases (correlation coefficient, R = 0.75). Higher levels of PAR1 expression were positively correlated with a worse prognosis in HER2-negative breast cancer. Thus, these results suggest that our new method to diagnose PAR1 expression levels by IHC with PAR1ab-QDs may facilitate clinical decisions in HER2-negative patients. Furthermore, anti-PAR1 antibody may become a critical molecular-targeting agent to improve the prognosis of breast cancer with PAR1 over-expression.

## Discussion

The monoclonal anti-PAR1 antibody specifically interacted with PAR1 on the cell membrane in PAR1-KPL cells and inhibited the PAR1-cleaving activity of MMP1. This inhibiting activity of anti-PAR1 antibody prevented the migration and invasion of PAR1-KPL cells. Thus, these data demonstrate the potency of anti-PAR1 antibody as a therapeutic agent. The levels of trastuzumab or pertuzumab in the blood are approximately 300–600 nM or 500–1,000 nM, respectively. Although 10 μM of monoclonal anti-PAR1 antibody was used in the cell migration and invasion assays, this dosage was higher than that of trastuzumab or pertuzumab. In addition to the monoclonal anti-PAR1 antibody, we developed a rabbit polyclonal anti-PAR1 antibody using the same antigen used for the monoclonal antibody. We investigated the effect of polyclonal anti-PAR1 antibody on the invasion activity of PAR1-KPL cells in the same manner as for the monoclonal antibody. The results show that the effect of 300 nM polyclonal anti-PAR1 antibody on invasion activity was similar to that of 10 μM monoclonal anti-PAR1 antibody (Supplementary Fig. 2). Therefore, humanization of the anti-PAR1 antibody and improvement of its association constant through genetic engineering may yield an effective anticancer drug against PAR1.

To investigate the effect of nanoparticles on cell migration, the biophysical response of cultured oral mucosa cells to titanium dioxide, silicon dioxide, and hydroxyapatite nanoparticles was recently examined by Tay *et al.*^30^,^31^, who showed that exposure to nanoparticles increases cell contractility, with significantly impaired wound healing (a cell migration activity) but no apparent cytotoxicity^30^,^31^. The underlying mechanism is thought to be interference of endocytosed nanoparticles with microtubule assembly and thus dysregulation of cell contractility and promotion of an adhesive phenotype^30^,^31^. In this study, using anti-PAR1-QD nanoparticles, we investigated the effect of PAR1 antibody on PAR1-cleaving activity by MMP1 and demonstrated that the binding of anti-PAR1-QDs and PAR1 on the cell membrane abrogates the PAR1-cleaving activity of MMP1. Accordingly, the effect of anti-PAR1 antibody on cell migration and invasion was examined. As we used anti-PAR1 antibody, rather than anti-PAR1-QDs, we believe that we do not need to consider the effect of nanoparticles on the migration and invasion, in contrast to the previous study by Tay *et al.*^30^.

Recent studies using cultured cells have demonstrated that PAR1 is highly expressed in invasive breast cancer and plays a critical role in metastasis in breast cancer. However, it has been difficult to measure the amount of PAR1 protein expressed in human breast cancer tissue using IHC-DAB because this method lacks quantitative sensitivity. In the present study, we developed a new IHC technique using an autofluorescence-subtracted image and single-QD imaging. This method has high quantitative sensitivity because positive QD signals are described based on the particle number as an absolute value, which is largely independent of the optical system or autofluorescence, unlike fluorescence intensity. Our imaging method defines single-QD fluorescence using the blinking reaction. Therefore, even if single QDs assemble locally within an area and their assembled QDs appear as a larger bright spot, we can estimate the particle number of QDs within the large spot with high accuracy. We used a 488-nm laser to obtain a subtracted image between the 640–690-nm and 695–740-nm images. The particle number of QDs in the autofluorescence-subtracted image in 532-nm (CrystaLaser) laser-excited tissues was highly similar to that in the 488-nm laser-excited tissues (Supplementary Fig. 3), which indicates that this method is effective with various excited wavelengths. These results show that our imaging method is user-friendly. When performing effective fluorescence imaging, autofluorescence or quantitative sensitivity is difficult for not only the pathologist but also other scientists performing bio-imaging. Thus, this method should be useful for many researchers.

In IHC with anti-PAR1-QDs using human breast cancer tissue samples with detailed clinical information, the number of QDs in a cancer cell was strongly correlated with the relapse-free survival time of HER2-negative breast cancer patients with metastasis within 3 years after surgery. The time to recurrence became progressively shorter in proportion to the number of anti-PAR1-QDs in the cancer tissues. Because our imaging method can exclude the possibility of a false-positive fluorescence signal derived from autofluorescence, we could determine the relationship between the PAR1 expression level and the prognosis of the patients. There are currently no markers to predict the prognosis of HER2-negative breast cancer patients. Therefore, PAR1 may be a promising marker to predict prognosis and make clinical decisions, and anti-PAR1 antibody may be an effective molecule-targeting agent to improve the prognosis of PAR1-expressing breast cancer patients (including HER2-negative cases). Recently, the concept of theranostics, agents that combine diagnosis and therapy, has received considerable attention. In this study, we showed that anti-PAR1 antibody might be useful for both diagnosis of the risk of breast cancer recurrence after surgery in HER2-negative and PAR1-positive patients and therapy for PAR1-positive patients as an anticancer drug acting against PAR1. Therefore, anti-PAR1 antibody shows promise as a theranostic agent for use in PAR1-positive cancer patients. Our imaging method can be applied to measure the expression levels of other proteins in various types of cancer. Therefore, this new method could be used to improve our understanding the mechanisms of cancer progression as well as the anti-tumor effects of anticancer agents.

## Methods

### PAR1 antibodies

In our previous study, a monoclonal anti-PAR1 antibody was prepared, and epitope mapping of the antibody identified its epitope as N_35_ATLDPRSFLL_45_, including the R_41_-S_42_ peptide bond that is cleaved by PAR1^24^. In the present study, we prepared polyclonal anti-PAR1 antibody using the same antigen, the oligopeptide CNATLDPRSFLL, to generate the monoclonal antibody. The oligopeptide was cross-linked with keyhole limpet hemocyanine, and the cross-linked oligopeptide was injected into a rabbit biweekly. After several injections, the antiserum was collected and purified with an affinity column using CNATLDPRSFLL oligopeptide.

### Preparation of anti-PAR1 antibody-conjugated QDs (anti-PAR1-QDs)

To obtain a highly pure monoclonal anti-PAR1 antibody, cloned hybridoma cells producing monoclonal anti-PAR1 antibody were injected into the abdominal cavity of a severe combined immunodeficient (SCID) mouse (Charles River), which is an immunodeficient mouse that lacks immunoglobulins. Ascites were prepared from the mouse, and anti-PAR1 antibody was purified from the ascites using protein G-Sepharose (Amersham Biosciences). The purified antibody was monomerized, mixed with QDs in a molar ratio of approximately 3:1 (antibody: QDs), and then applied to a preparation of anti-PAR1-QDs using a Qdot 705 Antibody Conjugation Kit (Life Technologies), where the number indicates the emission wavelength, as in our previous study^28^.

### Cultured cells

The human KPL-4 (KPL) breast cancer cell line^32^ was kindly provided by Dr. J. Kurebayashi (Kawasaki Medical School, Japan). In a previous study, KPL cells were transformed into metastatic cancer cell PAR1-expressing KPL (PAR1-KPL) cells by transduction with the pLNCX2 retroviral vector system, using the PAR1 gene as the insert. Then, the cells were cloned. KPL and PAR1-KPL cells were cultured in Dulbecco’s modified Eagle’s medium (Invitrogen) containing 10% fetal bovine serum (FBS). PAR1-KPL cells were grown in the presence of 400 g/ml G418.

### Binding assay of anti-PAR1-QDs to membrane-bound PAR1

To test the effect of anti-PAR1 antibody on the PAR1-cleaving activity of MMP1 (Sigma), three different experiments were performed using PAR1-KPL cells. First, PAR1-KPL cells were mixed with 2 μM control mouse IgG in serum-free L-15 medium (Invitrogen) for blocking for 15 min at 37 °C and then with 40 nM anti-PAR1-QDs in serum-free L-15 medium for 30 min at 37 °C. After being washed with L-15 medium, the cells were incubated with L-15 containing 0.5% FBS in a glass-bottomed dish and then observed. Second, PAR1-KPL cells were mixed with 2 μM control mouse IgG in serum-free L-15 medium for blocking for 15 min at 37 °C and then with 50 nM MMP1 in serum-free L-15 medium for 60 min at 37 °C. After they were washed with L-15 medium, the PAR1-KPL cells were incubated with 40 nM anti-PAR1-QDs in serum-free L-15 medium for 30 min at 37 °C. After being washed with L-15 medium, the cells were incubated with L-15 containing 0.5% FBS in a glass-bottomed dish and then observed. Third, PAR1-KPL cells were mixed with 2 μM control mouse IgG in serum-free L-15 medium for blocking for 15 min at 37 °C and then with 40 nM anti-PAR1-QDs in serum-free L-15 medium for 30 min at 37 °C. After they were washed with L-15 medium, the PAR1-KPL cells were incubated with 50 nM MMP1 in serum-free L-15 medium for 60 min at 37 °C. After they were washed with L-15 medium, the cells were incubated with L-15 containing 0.5% FBS in a glass-bottomed dish and the QD fluorescence signal was observed. In the above three experiments, the eleven cells for determination of the QD-fluorescence intensity in a cell were measured and calculated as gray values by subtracting the background values from the QD values.

### Cell migration assay

To test the effect of anti-PAR1 antibody on the migration activity of PAR1-KPL cells, three different experiments were performed. First, 7 × 10^4^ PAR1-KPL cells were treated with 10 μM control mouse IgG in L-15 medium for 60 min at 37 °C. Then, the cells were incubated with L-15 containing 10% FBS in a glass-bottomed dish and observed. Second, 7 × 10^4^ PAR1-KPL cells were treated with 10 μM control mouse IgG in L-15 medium for 60 min at 37 °C, incubated with 2.5 nM MMP1 in L-15 containing 10% FBS in a glass-bottomed dish, and then observed. Third, 7 × 10^4^ PAR1-KPL cells were treated with 10 μM monoclonal anti-PAR1 antibody in L-15 medium for 60 min at 37 °C, incubated with 2.5 nM MMP1 in L-15 containing 10% FBS in a glass-bottomed dish, and then observed. Time-lapse observation of each cell was performed every 2 min for 13 h. In each condition, the nuclear barycentric position of approximately 30 cells from 2–13-h video data were tracked using a previously described tracking method^33^. The nuclear position in each cell was calculated for every hour as the migration velocity (μm/h).

### Cell invasion assay

To investigate the effect of anti-PAR1 antibody on the invasion activity of PAR1-KPL cells, four different experiments were conducted using a Matrigel^TM^ invasion chamber (BD Bioscience). First, 1.5 × 10^5^ PAR1-KPL cells were treated with 10 μM control mouse IgG in L-15 medium for 90 min at 37 °C and placed in the cell-culture insert provided in the kit. The insert contains a matrigel-coated membrane filter with numerous pores (diameter 8 μm) at the bottom. The insert including the cells were placed in the well of the kit containing 10% FBS in L-15. Second, 1.5 × 10^5^ PAR1-KPL cells were treated with 10 μM control mouse IgG in L-15 medium for 90 min at 37 °C and placed in the cell-culture insert. The insert was place in the well containing 2.5 nM MMP1 in 10% FBS in L-15. Third, 1.5 × 10^5^ PAR1-KPL cells were treated with 3 μM monoclonal anti-PAR1 antibody in L-15 medium for 90 min at 37 °C and placed in the cell-culture insert. The insert was placed in the well containing 2.5 nM MMP1 and 10% FBS in L-15. Fourth, 1.5 × 10^5^ PAR1-KPL cells were treated with 10 μM monoclonal anti-PAR1 antibody in L-15 medium for 90 min at 37 °C and placed in the cell-culture insert. The insert was placed in the well containing 2.5 nM MMP1 and 10% FBS in L-15. In addition, to investigate the effect of polyclonal anti-PAR1 antibody on inhibition of the cell-invasion activity of PAR1-KPL cells, an experiment was performed substituting 300 nM control rabbit IgG, 100 nM polyclonal anti-PAR1 antibody, and 300 nM polyclonal anti-PAR1 antibody for 10 μM control mouse IgG, 3 μM monoclonal anti-PAR1 antibody, and 10 μM monoclonal anti-PAR1 antibody, respectively. In each of the above conditions, three Matrigel^TM^ invasion chambers were used, and the number of invaded cells in a 0.42-mm^2^ region on the bottom of the matrigel-coated membrane was counted. For each membrane of the Matrigel^TM^ invasion chambers, 10 points were measured.

### Optical Systems

Two types of optical systems were used in this study. One optical system observed the fluorescence of QDs and consisted primarily of an epi-fluorescence microscope (IX-71, Olympus) with modifications, a Nipkow disk-type confocal unit (CSU10, Yokogawa), and an electron multiplier-type charge-coupled device camera (EM-CCD, Ixon DV887, Andor Technology), which is a highly sensitive camera^24^,^34^,^35^. A PlanApo (X60, 1.40 NA, Olympus) objective lens was used for imaging. QDs were illuminated using a blue laser (488-nm wavelength, CrystaLaser). The laser-excited fluorescence was filtered with a 695–740-nm bandpass filter to image the QDs and autofluorescence of the tissues or a 640–690-nm bandpass filter to image the autofluorescence of tissues in an identical focal plate and field. Images were obtained at a rate of 5–10 frames per second. The other optical system was a Zeiss Pascal Confocal Microscope System (Zeiss). This system was used to observe cell movement for the cell migration assay or to measure the cell number for the cell-invasion assay.

### Human tissue samples and clinical information

We prepared the three datasets for the non-cancer cases and the HER2-negative breast cancer cases with and without metastasis. Surgical specimens of breast tissue were retrieved from four normal breast tissue samples of breast cancer patients, five breast cancer cases that remained non-metastatic for more than 5 years after surgery and eleven cases with metastasis within 3 years after surgery. The patients with metastasis were diagnosed with recurrence within 3 years after surgery at Tohoku University Hospital. Tissue samples with overexpression of human epidermal growth factor receptor 2 (HER2)/neu in their tumors were excluded to eliminate the influence of HER2/neu, which is one of the strongest prognostic factors in breast cancer. Clinical information, including the time from surgery to cancer metastasis, was collected from the breast cancer management database at Tohoku University.

### Ethics statement

The Ethical Committee of the Graduate School of Medicine, Tohoku University, approved the protocol. All of the patients signed an Ethical Committee consent form agreeing to serve as tissues donors for the experiments. The methods were carried out in “accordance” with the approved guidelines.

### Immunohistochemistry using anti-PAR1-QDs for human breast cancer

The specimens were fixed in 10% formalin, embedded in paraffin, cut into 3-μm-thick sections and placed on glue-coated glass slides. The sections were deparaffinized in xylene and hydrated with a graded alcohol series and distilled water. Antigen retrieval was performed using an autoclave (Tomy Sx-500 High Pressure Steam Sterilizer, Tomy Seiko Co., Ltd.) in 10 mM citrate buffer (pH, 6.0) heated to 121 °C for 15 min. To prevent non-specific binding of anti-PAR1 antibody, the samples were sequentially incubated with 50 mM NH_4_Cl in phosphate-buffered saline (PBS) at 25 °C for 30 min, 10% FBS in PBS at 25 °C for 60 min, and 10% FBS and 1 μM control mouse IgG in PBS at 25 °C for 3 h for blocking. Next, the samples were immunostained with 4.5 nM anti-PAR1-QDs or 4.5 nM control mouse IgG-conjugated QDs for 1.5 h at 25 °C. After washing with PBS, the samples were incubated with DAPI for nuclear staining. The samples were washed with PBS and mounted using mounting media (Aquatex, MERCK). The samples were observed with a single-particle imaging system.

### Comparison of the autofluorescence intensity and QD-fluorescence intensity

The change in the autofluorescence intensity at various wavelengths was investigated in human breast cancer tissues using various bandpass filters (505–545 nm, 565–595 nm, 585–630 nm, 640–690 nm, 695–740 nm, and 760–800 nm). The 488-nm laser-excited fluorescence intensity in an identical area of the tissues was measured. The fluorescence intensity at various wavelengths was divided by the wavelength interval. The change in QD fluorescence was obtained from the Life Technologies website. To compare single-particle QD fluorescence with autofluorescence, the fluorescence intensity in an arbitrary area (n = 15) in a region of interest (ROI) of the same size was measured using a 488-nm laser and a 695-740-nm bandpass filter. The three datasets for the change in autofluorescence intensity, the change in single-QD-fluorescence intensity, and the comparison of the autofluorescence and QD-fluorescence intensity were combined (Fig. 3a).

### Image analysis using single-particle QD imaging

To quantitatively measure the particle numbers of QDs bound to cancer tissues, analysis was conducted as follows: in 488-nm laser-excited tissues, 512-pixel square images, filtered with a 695–740-nm bandpass filter for QDs and tissue autofluorescence or a 640–690-nm bandpass filter for autofluorescence, were obtained at an exposure time of 20 s. Each image was converted into a JPEG file. During the conversion, the autofluorescence signal of the image filtered with a 640–690-nm bandpass filter (640–690-nm image) was adjusted to be approximately 1.2-fold greater than the signal filtered with the 695–740-nm bandpass filter (695–740-nm image). After file conversion, to allow us to visualize only the signal from the fluorescent QDs, the JPEG image of the 640–690-nm image was subtracted from that of the 695–740-nm image using Adobe Photoshop image-processing software (see Fig. 3). The fluorescence intensity of the QD signal in the subtracted image was analyzed as gray values using ImageJ software (http://rsb.info.nih.gov/ij/). The total fluorescence intensity in the image was defined as the total QD value. In the subtracted image, we also confirmed the fluorescence intensity in an area with an autofluorescence signal of zero (see Fig. 3). The gray value could not have a negative value. This result indicated that excluding the signal from the QDs, there was no fluorescent signal in the background of the subtracted images (see Fig. 3). To precisely measure the number of QD particles in the tissues, it was necessary to define the fluorescence intensity of a single QD. Because QDs possessing the same fluorescent wavelength are uniform in size, the fluorescence intensity of QDs is proportional to the particle number. In addition, the QD fluorescence consists of fluorescent and non-fluorescent states, called on and off states, respectively. This property results in blinking^36^. When we measured the fluorescence of fresh QD particles and analyzed their properties, the results indicated that the mean time spent in the off state during 20 s of observation was approximately 4 s, with a low s.e.m. value^37^. The mean time in the off state per unit time is shortened by aggregation of the QDs because the on and off states of each particle in the aggregate occur randomly. Therefore, based on an off-state time of 4 s, we selected a single-particle QD using each subtracted image and video image (see Fig. 4) and measured the fluorescence intensity of the single QD particle in the subtracted image (single QD value). In addition, the number of cells in each image was measured using the DAPI image (300–1,000 cells per patient sample). The total QD value was divided by the single QD value. Then, the number of QD particles was divided by the number of cells to calculate the number of QD particles in a cell. Finally, the value determined for the image labeled with QDs conjugated to control mouse IgG was subtracted from the value determined for the image labeled with anti-PAR1 QDs to obtain the precise mean number of QD particles that were specifically bound to a cancer cell.

### Statistical analysis

All of the data are presented as the mean ± s.e.m. An *F*-test was performed, and equal variance was defined as a *p*-value (*p*) ≧ 0.05. Comparisons between groups were performed using the parametric Student’s *t*-test (*p* ≧ 0.05 in the *f*-test) or Welch’s *t*-test (p < 0.05 in the *f*-test). *P* < 0.05 was considered significant for both *t*-tests.

## Additional Information

**How to cite this article**: Gonda, K. *et al.* Predictive diagnosis of the risk of breast cancer recurrence after surgery by single-particle quantum dot imaging. *Sci. Rep.* **5**, 14322; doi: 10.1038/srep14322 (2015).

## Supplementary Material

Supplementary Information

## Figures and Tables

**Table 1:** Results of immunostaining of pathological specimens with anti-PAR1-QDs.

	Recurrence	Sample No.	Age	Stage	HER2 Score	ER/PgR	Metastatic Site	Relapse-free survival time (years)	anti-PAR1-QDs score
Normal tissue		Sample 1	35						0.3
		Sample 2	47						0.1
		Sample 3	55						0.1
		Sample 4	56						0.2
Cancer tissue	Five years without recurrence	Sample 5	56	IIIA	0	+/+			4.8
		Sample 6	49	IA	1	+/+			5.1
		Sample 7	62	IIB	1	+/+			2.5
		Sample 8	41	IIB	1	+/+			2.1
		Sample 9	58	IIB	0	+/+			1.3
	Recurrence within 3 years	Sample 10	35	IIA	0	+/−	Lung	0.83	13
		Sample 11	47	IIIC	0	+/+	Bone, Liver	1.08	6.3
		Sample 12	51	IA	0	−/+	Brain, Lung, Lymph node,	0.83	25.2
		Sample 13*	40	IIB	1	−/−	Brain, Lung,	0.83	12.2
		Sample 14*	54	IIA	0	−/−	Brain, Liver, Lung	0.75	1.7
		Sample 15*	60	IIIC	1	−/−	Bone, Liver, Lymph node,	2	0.5
		Sample 16*	42	IIB	1	−/−	Brain, Lung	2	4
		Sample 17*	80	IA	0	−/−	Liver, Lung	0.67	5.5
		Sample 18*	75	IIIC	0	−/−	Bone, Liver	1.75	0.5
		Sample 19*	41	IA	1	−/−	Bone, Liver	2.58	0.5
		Sample 20*	55	IIIC	0	−/−	Brain, Liver, Lung	0.83	23.2

Four normal breast tissue samples from breast cancer patients (samples 1–4), five from HER2-negative breast cancer cases that remained non-metastatic for more than 5 years after surgery (samples 5–9), and 11 HER2-negative cases with metastasis within 3 years after surgery (samples 10–20) were selected. Asterisks indicate triple-negative cases (samples 13–20). The patients with metastasis were diagnosed as having a recurrence within 3 years after surgery at Tohoku University Hospital. The anti-PAR1-QD score shows the calculated number of QD particles in a cell. Based on these measurements, 300–1,000 cells were investigated per patient sample.
