# Supplementary material for: Predictive diagnosis of the risk of breast cancer recurrence after surgery by single-particle quantum dot imaging
Source: Sci Rep. 2015 Sep 22;5:14322. doi: 10.1038/srep14322 (PMC4585722; doi:10.1038/srep14322)
Supplement: Supplementary Information [file srep14322-s1.pdf]

Predictive diagnosis of the risk of breast cancer recurrence after surgery by single-particle quantum dot imaging

Kohsuke Gonda[1,2], Minoru Miyashita[3], Hideo Higuchi[4], Hiroshi Tada[3], Tomonobu M Watanabe[5], Mika Watanabe[6], Takanori Ishida[3], Noriaki Ohuchi[2,3].

[1]Department of Medical Physics, Graduate School of Medicine, Tohoku University, Seiryo-machi, Aoba-ku, Sendai 980-8575, Japan

[2]Department of Nano-Medical Science, Graduate School of Medicine, Tohoku University, Seiryo-machi, Aoba-ku, Sendai 980-8575, Japan

[3]Department of Surgical Oncology, Graduate School of Medicine, Tohoku University, Seiryo-machi, Aoba-ku, Sendai 980-8574, Japan

[4]Department of Physics, Graduate School of Science, University of Tokyo, Hongo Bunkyou-ku Tokyo 113-0033, Japan

[5]Laboratory for Comprehensive Bioimaging, RIKEN Quantitative Biology Center, OLABB, Osaka University, 6-2-3, Furuedai, Suita, Osaka 565-0874, Japan

[6]Department of Pathology, Tohoku University Hospital, Seiryo-machi, Aoba-ku, Sendai 980-8574, Japan

Supplementary figures and figure legends

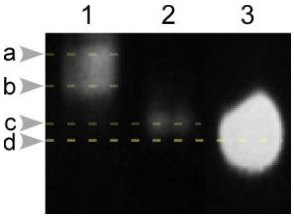

Supplementary Figure 1

The numbers of anti-PAR1 antibodies conjugated with single QD. To make the anti-PAR1-QDs used in this study, the monomerized monoclonal anti-PAR1 antibody and QDs were mixed in a molar ratio of approximately 3 : 1. Then, the anti-PAR1-QDs were prepared using a Qdot 705 Antibody Conjugation Kit (Life Technologies) according to the manufacturer's instruction. To estimate the number of monomer anti-PAR1 antibody bound to the surface of single QD, the anti-PAR1-QDs was separated by 0.8% agarose-gel electrophoresis, and then QDs fluorescence intensity of each band was calculated using ImageJ software. The sample of anti-PAR1-QDs was fractionated by the electrophoresis into two major bands. Approximately 52% of the anti-PAR1-QDs was conjugated with three monomer antibody fragments (lane 1, band a) and 48% with two fragments (lane 1, band b). The mean value was 2.5 monomer antibody fragments per QD. Band c in lane 2 shows the anti-PAR1-QDs conjugated with a single fragment. To prepare the anti-PAR1-QDs conjugated with a single fragment, anti-PAR1 antibody and QDs were mixed in a molar ratio of approximately 1 : 1; then, anti-PAR1-QDs was made using the kit. The prepared sample was analyzed by 0.8% agarose-gel electrophoresis (lane 2). The binding ability of anti-PAR1-QDs conjugated with a single fragment to PAR1 was weaker than that of anti-PAR1-QDs conjugated with 2.5 fragment per QD (data not shown). Therefore, anti-PAR1-QDs conjugated with a single fragment was not suitable for immunostaining with anti-PAR1-QDs. Band d in lane 3 shows the QDs without antibody fragments conjugated. The dotted lines show the center position of each band. This data was similar to the result in our previous study[28].

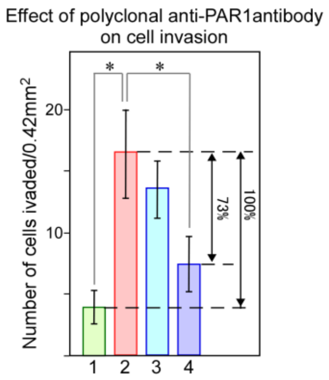

Supplementary Figure 2

The effect of polyclonal anti-PAR1 antibody on PAR1-KPL cell invasion activated by MMP1. Bar 1, the number of invading PAR1-KPL cells in a 0.42-mm^2 area treated with 300 nM control rabbit IgG. Bar 2, the number of invading PAR1-KPL cells in a 0.42-mm^2 area treated with 300 nM control rabbit IgG and then 2.5 nM MMP1. Bar 3, the number of invading PAR1-KPL cells in a 0.42-mm^2 area treated with 100 nM polyclonal anti-PAR1 antibody and then 2.5 nM MMP1. Bar 4, the number of invading PAR1-KPL cells in a 0.42-mm^2 area treated with 300 nM polyclonal anti-PAR1 antibody and then 2.5 nM MMP1. The following calculation was performed to estimate the inhibition of cell invasion by treatment with 300 nM anti-PAR1 antibody. This figure shows that PAR1 antibody in the presence of MMP1 results in a decrease in the number of cells (18.8 cells [bar 2] - 8.0 cells [bar 4] = 10.8 cells/0.42 mm^2) compared with MMP1 alone, which increases the number of cells (18.8 cells [bar 2] - 4.1 cells [bar 1] = 14.7 cells/0.42 mm^2) was 73% (10.8/14.7 x 100%), demonstrating that the PAR1 antibody inhibited the cell invasion activated by MMP1.

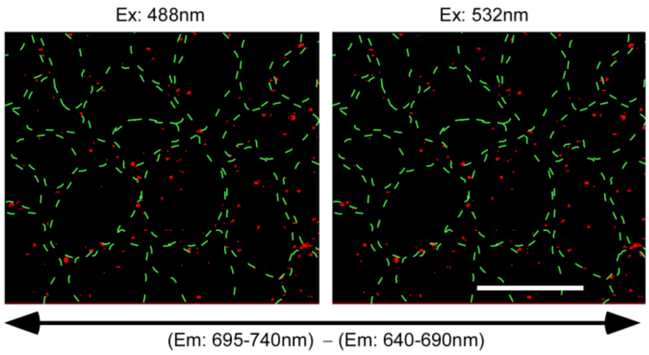

Supplementary Figure 3

Comparison of the autofluorescence-subtracted image in 488-nm laser-excited tissue with that in 532-nm laser-excited tissue. In this study, a 488-nm laser was mainly used for excitation to obtain a subtracted image between the 640-690 and 695-740-nm images. The particle number of QDs (red spots) in the autofluorescence-subtracted image of 532-nm laser-excited tissues was similar to that in the autofluorescence-subtracted image of 488-nm laser-excited tissues. The green dotted lines indicate the cellular outline determined using a bright-field image. Bar, 10 μm.
